# Recent Advances in Lipid Nanoparticles and Their Safety Concerns for mRNA Delivery

**DOI:** 10.3390/vaccines12101148

**Published:** 2024-10-08

**Authors:** Jialiang Wang, Yaopeng Ding, Kellie Chong, Meng Cui, Zeyu Cao, Chenjue Tang, Zhen Tian, Yuping Hu, Yu Zhao, Shaoyi Jiang

**Affiliations:** 1 Meinig School of Biomedical Engineering, Cornell University, Ithaca, NY 14853, USA; 2 Smith School of Chemical and Biomolecular Engineering, Cornell University, Ithaca, NY 14853, USA; kc2246@cornell.edu (K.C.);; 3 Department of Materials Science and Engineering, Cornell University, Ithaca, NY 14853, USA; 4 Department of Biological and Environmental Engineering, Cornell University, Ithaca, NY 14853, USA

**Keywords:** lipid nanoparticle, selective organ-targeting LNP, endosomal escape, toxicity, reactogenicity, immunogenicity

## Abstract

Introduction: The advent of lipid nanoparticles (LNPs) as a delivery platform for mRNA therapeutics has revolutionized the biomedical field, particularly in treating infectious diseases, cancer, genetic disorders, and metabolic diseases. Recent Advances in Therapeutic LNPs: LNPs, composed of ionizable lipids, phospholipids, cholesterol, and polyethylene glycol (PEG) lipids, facilitate efficient cellular uptake and cytosolic release of mRNA while mitigating degradation by nucleases. However, as synthetic entities, LNPs face challenges that alter their therapeutic efficacy and safety concerns. Toxicity/Reactogenicity/Immunogenicity: This review provides a comprehensive overview of the latest advancements in LNP research, focusing on preclinical safety assessments encompassing toxicity, reactogenicity, and immunogenicity. Summary and Outlook: Additionally, it outlines potential strategies for addressing these challenges and offers insights into future research directions for enhancing the application of LNPs in mRNA therapeutics.

## 1. Introduction

In 1990, Wolff et al. first demonstrated that intramuscular injection of mRNA into the skeletal muscle of mice resulted in the expression of encoded proteins, laying a foundational concept for mRNA therapeutics [1]. In 2005, scientists Katalin Karikó and Drew Weissman discovered that modifying nucleosides in mRNA could significantly reduce the immune response, enhancing the viability of mRNA for therapeutic use [2]. mRNA technology has since shown immense potential in treating various diseases, revolutionizing infectious diseases, cancer, genetic disorders, and metabolic diseases [3]. It offers speed, flexibility, high efficacy, and safety by not integrating into the host genome. However, the inherent instability of mRNA necessitates a packaging and delivery system to protect it from nuclease degradation and ensure efficient cellular uptake, intracellular release, and translation into protein [4]. Consequently, a delivery system is essential to enhance the absorption efficiency of targeted cells and tissues, allowing the mRNA to reach the therapeutic threshold without administering excessively high doses of naked mRNA, which can lead to cytotoxicity and immune responses. Following the outbreak of COVID-19 at the end of 2019, there was an urgent global need for a safe and effective vaccine to combat this highly contagious virus. This demand significantly increased investment from governments and the private sector and fostered global cooperation among scientists, researchers, pharmaceutical companies, and governments. Therefore, mRNA vaccines have transitioned from the laboratory to widespread public attention. A significant milestone is the integration of lipid nanoparticles (LNPs) and mRNA technologies. In clinical practice, researchers have demonstrated the therapeutic efficacy of mRNA by incorporating LNPs [5]. Notably, the two FDA-approved mRNA vaccines utilize LNPs as the carriers of mRNA [6]. To further extend the application of LNPs in the biomedical field, it is crucial to gain a comprehensive understanding of their properties and develop a robust evaluation system to assess their effectiveness and safety.

**Figure 1:**
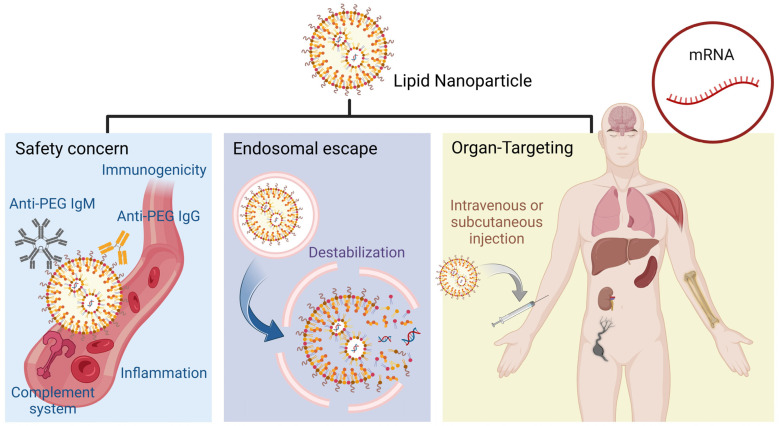
Lipid nanoparticles for organ-targeting mRNA delivery and their safety concerns for mRNA delivery. (Figure created with BioRender.com).

LNPs are derived from the phospholipid-based liposomes developed in the 1960s [7]. By the 1970s, the addition of cholesterol to liposomes was reported to enhance their stability during in vivo delivery [8]. In the 1980s, to increase the loading capacity of negatively charged mRNAs in liposomes, researchers began adding positively charged cationic lipids to existing formulations [9]. While cationic lipids successfully increased mRNA loading capacity, they introduced significant drawbacks, including cytotoxicity, opsonization with plasma proteins, and low transfection efficiencies due to rapid splenic and hepatic clearance [10]. To mitigate these issues, ionizable lipids and Polyethylene glycol (PEG)-ylated lipids were incorporated into LNP formulations. Ionizable lipids remain neutral in circulation, avoiding cellular or molecular recognition, but become protonated and fuse with the endosomal membrane after cellular uptake, releasing the mRNA cargo into the cytoplasm for translation [11]. PEG enhances the colloidal stability of nanoparticles in fluids and is physiologically inert, cloaking potential epitopes, thus reducing aggregation and opsonization while improving the immunogenicity and in vivo retention of PEGylated LNPs, enhancing safety and efficacy [12]. Therefore, current LNPs typically comprise four main components: ionizable lipids, which bind to negatively charged cargoes and assist in endosomal escape; phospholipids, which provide structural integrity; cholesterol, which enhances nanoparticle stability and facilitates membrane fusion; and PEGylated lipids, which improve nanoparticle stability and circulation [13]. Based on the development over several decades, LNP has become the most versatile platform for mRNA in vivo organ-targeting delivery. However, LNPs, as synthetic delivery systems created in the lab, are foreign according to the immune system, which influences their effectiveness and safety. Their potential for toxicity arises from lipid composition, where ionizable lipids may interact with Toll-like receptors (TLRs), posing risks for inflammatory response [14]. Reactogenicity includes local reactions such as pain or redness at the injection site and systemic responses such as fever, driven by the body’s immune response to both the LNPs and their cargoes [15]. Inflammatory responses may occur due to interactions between LNPs and immune cells, and while a controllable extent of inflammation is necessary for efficacy, excessive inflammation can be detrimental. Immunogenicity involves the LNPs’ ability to provoke an immune response through their lipid components, mRNA delivery, and antigen presentation, with a careful balance required to avoid overreaction [16]. The overall effectiveness and safety of LNPs depend on meticulous design and evaluation to manage these factors, ensuring they remain a safe and effective tool for therapeutic and vaccine applications (Figure 1).

This review includes the recent advancements in the field of LNPs over the past five years. Significantly, it provides an in-depth analysis of a crucial preclinical milestone for LNPs: the safety assessment encompassing toxicity, reactogenicity, and immunogenicity. Additionally, the review discusses potential solutions and future research directions, aiming to guide the future development of LNPs.

## 2. Recent Advances in Therapeutic LNPs

Ionizable lipids improve the encapsulation, stability, and delivery of mRNAs. These lipids possess unique pH-responsive properties that enable them to remain neutral during systemic circulation and become positively charged in acidic environments, such as the endosomes [17,18]. Modifying the pK_a_ of ionizable lipids influences transfection efficiency. Specifically, at physiological pH, approximately 7.4, these lipids are neutral, promoting stable LNP formation, extending circulation in the bloodstream, and preventing clearance by immune cells. In the acidic environment of endosomes, where pH ranges from 5.5 to 6.3, ionizable lipids become protonated, gaining a positive charge. This charge switch of ionizable lipids facilitates the endosomal membrane disruption by directly interacting with the negatively charged phospholipids, enhancing the cargo release into the cytoplasm [19]. In addition, Dahlman et al. demonstrated that changing the stereochemistry of ionizable lipids, such as chirality, can also improve the delivery efficiency [20]. By synthesizing and testing 128 novel LNPs containing stereopure and racemic derivatives of the ionizable lipid C12-200, these researchers found that stereopure C12-200-S LNPs delivered up to 6.1-fold more mRNA in vivo than racemic and C12-200-R controls. This study highlights the potential of stereochemistry in optimizing LNP-mediated delivery systems for therapeutic applications. Another determinant factor in stereochemistry is the number of ionizable lipid tails, where multi-tail structures are cone-shaped, increasing the cross-section of the hydrophobic region and enhancing endosomal membrane disruption. Further discussions are provided in Section 2.2.

Regarding the LNP structural stability, cholesterol remains the overall shape by modulating the fluidity and permeability of the lipid bilayer, which is essential for effective encapsulation and protection of the mRNA cargoes [21]. Recent studies have been focused on optimizing cholesterol derivatives to improve LNP performance. Modified cholesterol molecules increase the stability and circulation time of LNPs, leading to enhanced delivery efficiency of mRNAs. Patel et al. investigated the incorporation of hydroxycholesterols into LNPs as a novel strategy to overcome delivery barriers such as endosomal recycling [22]. This research demonstrated that substituting 25% and 50% of cholesterol with 7α-hydroxycholesterol significantly improved mRNA delivery efficiency by 1.8-fold and 2.0-fold in primary human T cells ex vivo. This improvement is attributed to the modified LNPs’ ability to increase late endosome production and reduce recycling endosomes, thereby facilitating better endosomal escape of cargo. The findings underscore the potential of hydroxycholesterol modifications in LNP formulations to optimize mRNA delivery systems, which could have profound implications for developing advanced immunotherapies and vaccines. Choi et al. also improved the delivery efficiency, but in a different way [23]. They replaced cholesterol with 3β[L-histidinamide-carbamoyl] cholesterol (Hchol), improving the mRNA delivery and gene expression both in vitro and in vivo compared to regular LNPs depending on the pH-sensitive protonation of the imidazole groups in Hchol formulations. Modifications of cholesterol charges also play a significant role in targeting. Introducing cationic cholesterol into LNP substantially influences the delivery ratio between different organs [24]. For instance, LNPs composed of cationic cholesterol alter the particle tropism, targeting the lungs and heart more than traditional LNPs do.

Phospholipids are critical components due to their ability to enhance stability, facilitate mRNA encapsulation, and promote cellular uptake. Phospholipids, such as 1,2-distearoyl-sn-glycero-3-phosphocholine (DSPC) and 1,2-dioleoyl-sn-glycero-3-phosphoethanolamine (DOPE), have a unique truncated cone-like shape due to the bends in their acyl chains caused by double bonds, and they possess a smaller headgroup compared to the bulkier trimethylated amine group in phosphatidylcholine (PC). This distinct structure enhances the delivery of mRNAs into cells, facilitating membrane fusion between LNPs and cell membranes. Lastly, the primary functions of PEGylated lipid include enhancing the stability and circulation time of LNPs in the bloodstream, thereby reducing rapid clearance by the mononuclear phagocyte system (MPS). This improves the delivery efficiency of encapsulated therapeutic agents to target sites [25]. Recent studies have demonstrated that modifying the PEG chain length, architecture, and lipid fragment composition can significantly influence the performance of LNPs, optimizing them for specific therapeutic purposes. Recently, the immunogenicity of PEG has drawn attention due to the potential development of anti-PEG antibodies after repeated exposure. These antibodies can cause allergic reactions and induce an accelerated blood clearance (ABC) effect that impacts the therapeutic efficacy of repeated dosing, which will be further analyzed in Section 5. In recent years, numerous organ-targeting LNPs have been reported for mRNA therapy, and these advanced LNPs will be discussed in detail in the next section (Table 1).

### 2.1. Ionizable Lipids for Constructing Selective Organ-Targeting LNPs

**Figure 2:**
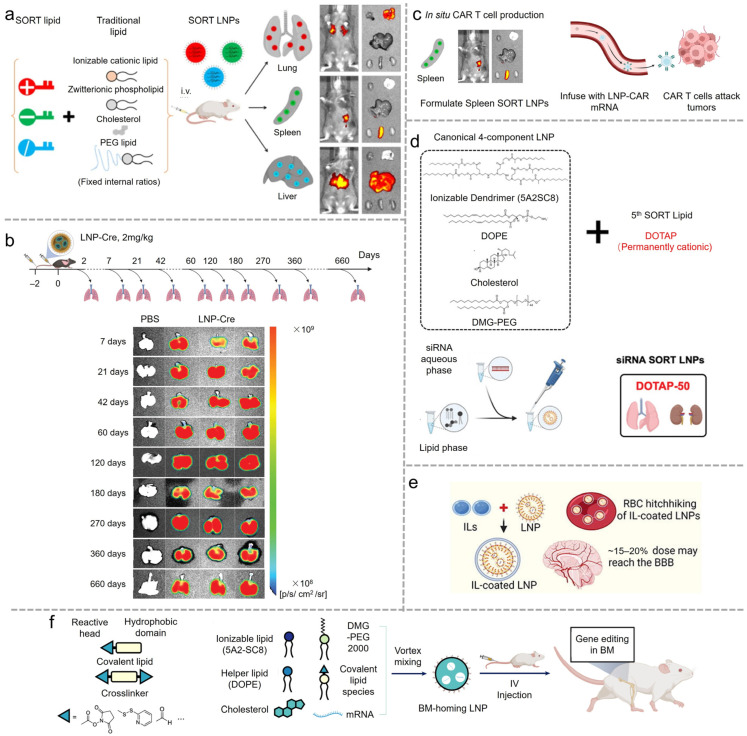
(**a**) Preparation of SORT LNPs using multiple technical methods for tissue-specific mRNA delivery. Reproduced from ref. [29]. Copyright Nature Publishing Group. (**b**) In vivo editing of lung stem cells for durable gene correction in mice. Reproduced from ref. [30]. Copyright AAAS. (**c**) Spleen SORT LNP-generated in situ CAR T cells extend survival in a mouse model of lymphoreplete B cell lymphoma. Reproduced from ref. [31]. Copyright Wiley-VCH. (**d**) Expanding RNAi to kidneys, lungs, and spleen via SORT siRNA LNPs. Reproduced from ref. [28]. Copyright Wiley-VCH. (**e**) LNP-mediated drug delivery to the brain. Reproduced from ref. [40]. Copyright Elsevier. (**f**) Bone-marrow-homing LNPs for genome editing in diseased and malignant hematopoietic stem cells. Reproduced from ref. [26]. Copyright Nature Publishing Group.

The default accumulation site of LNPs is the liver, where they acquire apolipoprotein E (apoE) from the bloodstream, facilitating their uptake by hepatocytes through interaction with the low-density lipoprotein receptor (LDLR) [37,38]. Alternatively, LNPs can also be decorated with N-acetylgalactosamine (GalNAc) ligands, which bind to the asialoglycoprotein receptor (ASGPR) on hepatocytes, directing the nanoparticles through receptor-mediated endocytosis [37]. This dual approach maximizes therapeutic efficacy and minimizes off-target effects, offering a robust framework for liver-specific delivery of advanced LNP-based therapies. Nevertheless, commercial LNP formulations have primarily been used for intramuscular (IM) administration, such as in COVID-19 mRNA vaccines, targeting the liver. This narrow focus constrains their potential applications beyond liver-targeted treatments. Targeting organs beyond the liver is critical due to the potential to enhance therapeutic efficacy for different diseases and reduce hepatic toxicity. LNPs can also concentrate therapeutic agents in targeted tissues by enabling organ-specific drug delivery. Siegwart et al. developed the Selective Organ Targeting (SORT) system, directing LNPs to the lung, spleen, and liver after intravenous (IV) administration [29] (Figure 2a). By introducing SORT molecules based on the four existing lipids mentioned above, the physicochemical properties of the nanoparticles are modulated. SORT molecules include cationic lipids for lung targeting, anionic lipids for spleen targeting, and ionizable amino lipids for liver targeting. These lipids interact with the traditional components, altering the nanoparticles’ surface charge, hydrophobicity, and interaction with biological membranes. As a result, LNPs can engage with specific cellular receptors, promoting selective uptake by target cells through receptor-mediated endocytosis or enhanced membrane fusion. In recent advancements, they presented a gene editing approach based on the SORT system, achieving significant and long-lasting genome editing in lung stem cells after IV administrations [30]. These researchers achieved over 70% editing efficiency in lung stem cells, with sustained expression in more than 80% of lung epithelial cells for 660 days (Figure 2b). This work not only highlights the potential of LNP-mediated gene editing to produce durable therapeutic effects for genetic lung diseases but also signifies a major advancement in overcoming the challenges associated with targeting tissue-resident stem cells. Other respiratory lung diseases can be accomplished by nebulized mRNA LNPs, which increase the LNP accumulation at the target tissue and avoid clearance when traveling through physiological barriers [39]. Traditional LNPs primarily accumulate in non-immune organs such as the liver and lungs, triggering toxic reactions or inflammatory responses. Targeting the spleen can minimize off-target effects due to its role as a key organ in the immune system, particularly in the regulation and production of immune cells. The SORT system was applied to deliver Cre recombinase mRNA and Chimeric Antigen Receptor (CAR) encoding mRNA to both CD4^+^ and CD8^+^ T cells without requiring active targeting ligands after IV administration [31] (Figure 2c). Moreover, Siegwart et al. expanded applications of the SORT system, delivering LNPs to kidneys [28]. These researchers optimized lipid compositions, with specific emphasis on the inclusion of supplemental SORT lipids such as DOTAP. These lipids significantly influence the biodistribution of the LNPs, enabling a substantial fraction to reach the kidneys. The DOTAP-50 LNP formulation, for instance, has shown remarkable efficiency, with approximately 13% of the administered dose accumulating in the kidney tissues (Figure 2d). This targeted delivery facilitates effective gene silencing, demonstrated by the significant knockdown of the Tie2 gene, which plays a critical role in maintaining vascular integrity.

Modifying LNPs with specific ligands or antibodies that bind to specific cell receptors facilitates precise delivery to other organs that are difficult to target. Brain targeting is significant in treating central nervous system (CNS) disorders such as Alzheimer’s, Parkinson’s, and brain tumors, which are challenging due to the protective nature of the blood-brain barrier (BBB). The mechanism of brain targeting with LNPs involves surface modification with targeting ligands such as transferrin or lactoferrin, which facilitate receptor-mediated transcytosis across the BBB upon IV injections [40,41]. An alternative mechanism for brain targeting involves coating LNPs with ionic liquids (ILs), such as choline carboxylates, to enable red blood cell (RBC) hitchhiking [42,43]. This process begins by coating LNPs with ILs, facilitating their adhesion to RBC membranes upon IV injection (Figure 2e). The IL-coated LNPs hitch a ride on circulating RBCs, which naturally navigate through the bloodstream to the brain due to the high perfusion of the brain tissue. Once the RBCs reach the brain’s microvasculature, the shear forces and interactions with endothelial cells cause the IL-coated LNPs to detach from the RBCs. The detached LNPs can then cross the BBB via receptor-mediated transcytosis or other transport mechanisms. Additionally, a recent achievement in targeting bone marrow was achieved by integrating a commercial formulation of covalent-bond-forming lipid species, enabling high transfection efficiency across multiple hemopoietic stem cells after IV injection [26] (Figure 2f). The fulfillment of ApoE on the LNP surface was crucial for bone marrow homing. Mitchell et al. also accomplished bone targeting by designing bisphosphonate (BP) lipid-like materials, demonstrating a high affinity for bone minerals to enhance the delivery of mRNAs to the bone microenvironment [27].

**Figure 3:**
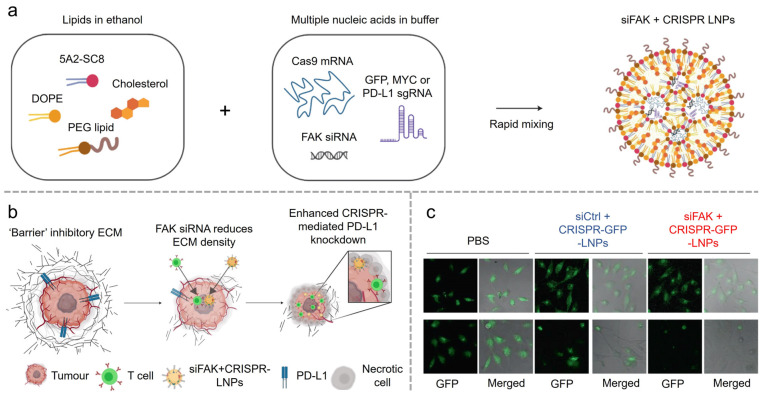
(**a**) Enhancing CRISPR/Cas gene editing through modulating cellular mechanical properties for cancer therapy. Illustration of triple loading of FAK siRNA, Cas9 mRNA, and sgRNA into 5A2-SC8 LNPs. (**b**) Dendrimer LNPs encapsulating FAK siRNA, Cas9 mRNA, and targeted sgRNAs could exhibit enhanced penetration into tumors with increased gene editing of PD-L1 for improved cancer therapy. (**c**) Representative fluorescence microscopy images at 0 h (top) and 48 h (bottom) and quantification of GFP fluorescence intensity. Reproduced from ref. [44]. Copyright Nature Publishing Group.

LNPs have significantly advanced the therapeutic potential of gene-editing technologies. A key challenge in the application of the CRISPR-Cas9 system is the efficient delivery of all components into target cells. Siegwart et al. provided an exemplary demonstration of mRNA delivery for cancer therapy [44]. They developed a multiplexed nanoparticle system designed to encapsulate siRNA, Cas9 RNA, and sgRNA (Figure 3). This approach effectively reduced tumor rigidity, enhanced nanoparticle endocytosis and tissue penetration, and lowered the therapeutic modification threshold, thereby enabling gene-editing therapies to confer a substantial survival advantage in genetically engineered mouse models of aggressive tumors. Furthermore, CRISPR-Cas9, with organ-specific targeting, has demonstrated potential for in vivo gene correction in disease models. These researchers from the same group also showed the efficacy of their treatment in a cystic fibrosis mouse model, where lung-specific LNPs facilitated the accumulation of gene-editing components in the lungs [45]. Following in vivo gene editing, the CFTR function in mutant mice was restored, providing a promising therapeutic outcome. The targeted strategies outlined above represent key efforts by researchers to address safety concerns associated with LNPs at this stage.

### 2.2. Robust Cytosolic Delivery through Improving Endosomal Escape

Although 95% of LNPs are taken up by cells, less than 2% of mRNAs delivered by LNPs successfully escape the endosomes and reach the cytoplasm [46,47]. The low efficiency of endosomal escape is due to cargo not being released in the early endosome and then being transferred to the late endosome and lysosome, where it is prone to degradation. This entrapment restricts mRNAs from successfully escaping the endosomal compartment and reaching the cytoplasm, limiting the overall effectiveness of mRNA-LNPs [48]. LNPs are either taken up by clathrin-mediated endocytosis or micropinocytosis and then transferred to the early and late endosomal compartments for mRNA release [49]. A consensus is lacking regarding the exact endosomal compartment where mRNAs are released from LNPs. Zerial et al.’s studies suggest mRNA is released from a hybrid compartment with characteristics of both early and late endosomes, while Anderson et al. believe in releasing the cargo in the late endosomal compartment [46,50].

**Figure 4:**
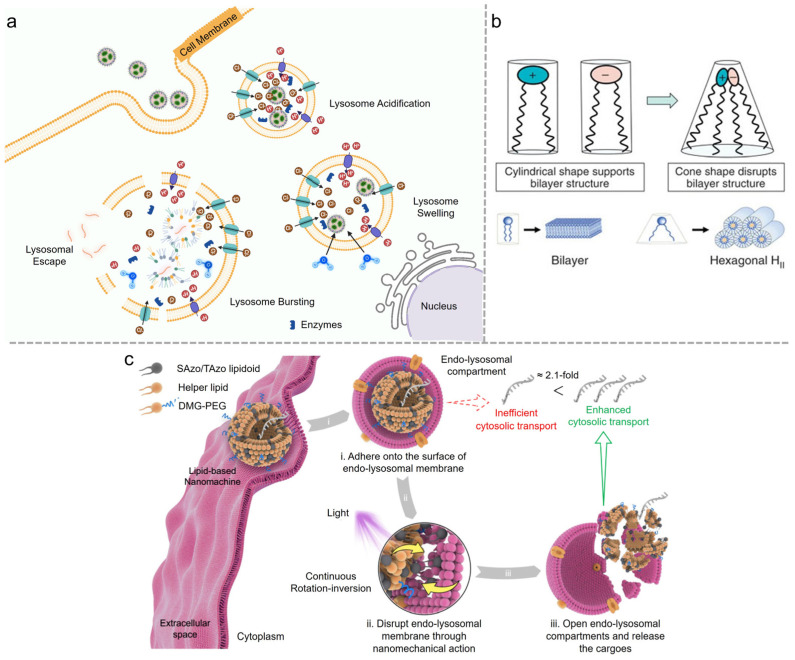
(**a**) Endosomal escape: A bottleneck for LNP-mediated therapeutics. Reproduced from ref. [48]. Copyright NAS. (**b**) Rational design of cationic lipids for siRNA delivery. Reproduced from ref. [11]. Copyright Nature Portfolio. (**c**) Nanomechanical action opens endo-lysosomal compartments. Reproduced from ref. [32]. Copyright Nature Portfolio.

A prevalent theory regarding endosomal escape is the proton sponge effect. This effect significantly depends on the buffering capacity of ionizable lipids after LNPs are endocytosed [48]. Ionizable lipids maintain a neutral pH at physiological conditions and get protonated after being internalized into the acidic endosomal compartment [51]. The acidic environment in endosomes allows ionizable lipids to be protonated and activate proton pumps, increasing their membrane potential [52]. To balance the rising charge, chloride ions enter the endosome, increasing the osmotic pressure, causing the endosome to swell and eventually rupture so LNP cargo can be released into the cytoplasm (Figure 4a). Another well-known endosomal escape theory involves membrane disruption. Ionizable lipids electrostatically interact with negatively charged anionic lipids in the endosomal membrane. Due to their larger headgroups and smaller hydrophobic tails, the ion pairs form a cone shape, creating inverted non-bilayer structures known as the hexagonal H_II_ phase [11] (Figure 4b). This structural transition disrupts the endosomal membrane, causing its destabilization and releasing the mRNAs into the cytoplasm.

Leal et al. proposed the topology of LNPs, especially the organization of nucleic acids and lipids within the particles, determining membrane fusion and affecting endosomal escape [53]. LNPs with lamellar structures face challenges in achieving efficient fusion due to the higher energy barriers they encounter, whereas LNPs with cuboplex or inverse hexagonal phases overcome these barriers more easily. These LNPs possess periodic internal structures, enabling them to form fusion pores when interacting with endosomal membranes and therefore enhance endosomal escape. Another challenge is the inefficiency of intracellular delivery of LNPs since they are trapped in endo-lysosomal compartments and unable to reach the cytoplasm after being endocytosed. This difficulty was solved by Xu et al., who presented a lipid-based nanoscale molecular machine (LNM) composed of photoisomerable amphiphilic azobenzene (Azo)-based lipidoids and helper lipids [32] (Figure 4c). LNM undergoes continuous rotation-inversion movements following light irradiation, destabilizing and disrupting the endo-lysosomal membrane so the cargo can be released into the cytoplasm [54]. Current research proposes inflammation induced by the release of Cathepsin B/D after the disruption of the endosome, triggering inflammation and subsequent side effects, which will be further analyzed in Section 3 [55,56]. In fact, improving endosomal escape efficiency enhances drug bioavailability, allowing for reduced dosages. This is also an effective strategy for mitigating potential safety concerns associated with mRNA-LNPs.

## 3. Toxicity

Ionizable lipids, key components of LNPs, play crucial roles in enhancing the delivery and efficacy of facilitating endosomal escape and cytosolic release of mRNAs. However, their potential toxicity must be scrutinized due to their involvement in cellular signaling, energy metabolism, and immunity. They can activate TLRs, particularly TLR4, leading to the production of pro-inflammatory cytokines such as interleukin-6 (IL-6), C-C motif chemokine ligand 2 (CCL2), and C-X-C motif chemokine ligand 2 (CXCL2) [57]. Such an immunostimulatory effect has been observed with ionizable lipids such as DLin-MC3-DMA and C12-200. Metabolites, such as fatty acids, derived from ionizable lipids induce toxicity by activating peroxisome proliferator-activated receptors (PPARs). Activation of these pathways can result in inflammation and liver toxicity [14]. For instance, empty LNPs containing the ionizable lipid YSK13 have been shown to elevate plasma levels of alanine aminotransferase (ALT) and aspartate aminotransferase (AST), markers of liver injury, through hepatic neutrophil infiltration [58]. Another source of toxicity is PEGylated lipids, where the long-term safety of PEGylated lipids is a concern due to their potential to alter the pharmacokinetics and biodistribution of LNPs [14]. Repeated administration of PEGylated LNPs can provoke an immune response, leading to the production of anti-PEG antibodies. These antibodies can rapidly clear subsequent doses of PEGylated LNPs from the bloodstream via accelerated blood clearance [59]. This not only diminishes the therapeutic efficacy of the treatment but also increases the risk of adverse reactions due to the rapid and unexpected distribution of the nanoparticles. Except for lipids, lysosomal cysteine proteases, such as Cathepsin B/D, can also induce inflammation and toxicity. These proteases are released into the cytosol during lysosomal membrane permeabilization (LMP) after LNPs are endocytosed, causing inflammation due to the activation of the NLRP3 inflammasome and further facilitating the release of pro-inflammatory cytokines [55]. Cathepsins also lead to cellular toxicity by promoting cell death and necroptosis by activating apoptosis pathways and rupturing the plasma membrane [60,61]. The cargo of LNP, mRNA, can cause off-target effects, innate immune activation, and protein overexpression, leading to toxicities as well. Off-target delivery results in non-antigen-presenting cells expressing antigens, triggering unintended immune responses and tissue-specific damage, such as myocarditis. Also, mRNA is inherently unstable and can easily be degraded, releasing fragmented RNA [14]. These fragments can act as DAMPs, triggering further immune activation and toxicity and therefore inducing excessive cytokine release and systemic inflammation.

**Figure 5:**
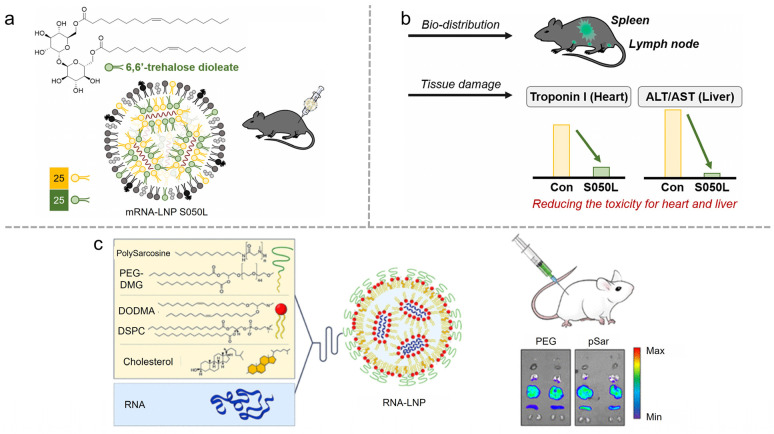
(**a**,**b**) A LNP platform incorporating trehalose glycolipid for exceptional mRNA vaccine safety. Reproduced from ref. [33]. Copyright KeAi Communications Co., Ltd. (**c**) Polysarcosine-functionalized LNPs for therapeutic mRNA delivery. Reproduced from ref. [34]. Copyright ACS.

A solution to reduce LNP toxicity is to decrease the amount of ionizable lipid, replacing it with a biodegradable component. Bang et al. substitute ionizable lipids with trehalose glycolipids to reduce toxicity commonly associated with conventional LNP formulations [33]. Modifying the lipid head with 6,6′-trehalose dimycolate allows the LNPs to break down into non-toxic metabolites after delivering their cargoes (Figure 5a). Comparative studies showed that LNPs containing trehalose glycolipids (LNP S050L) exhibited significantly lower toxicity in various organs, including the heart and liver, than conventional LNPs. LNP S050L maintained equivalent immunogenicity, indicating their potential for effective mRNA delivery (Figure 5b). Another approach is to replace PEGylated lipids with Polysarcosine (pSar) derived from the endogenous amino acid sarcosine, which reduces proinflammatory cytokine secretion and lowers complement activation, mitigating the risks of hypersensitivity reactions and complement activation-related pseudo-allergy (CARPA) [34] (Figure 5c). This PEG-free approach facilitates higher protein expression with an improved safety profile, making pSar-functionalized LNPs a promising platform with reduced toxicity.

## 4. Reactogenicity

Vaccine reactogenicity is the physical manifestation of the inflammatory response triggered by a vaccine, encompassing both local and systemic reactions. Common local side effects include swelling, redness, pain, and heat at the injection site, which are more prevalent in recipients of mRNA vaccines compared to those receiving a placebo. Systemic side effects, such as fatigue, headache, fever, myalgia, and arthralgia, are also more common following mRNA vaccination, with increased severity typically observed after the second dose, particularly in younger individuals (16–55 years) compared to older adults (over 55 years) [62]. Although serious adverse events such as acute myocardial infarction, Bell’s palsy, cerebral venous sinus thrombosis, Guillain–Barré syndrome, myocarditis/pericarditis, pulmonary embolism, stroke, and anaphylaxis are rare, they can be significant. Anaphylaxis, for example, is caused by PEGylated lipids, where the production of cytokines, such as IL-1 and IL-6, plays a pivotal role in the immune response and is closely associated with the reactogenicity profile of vaccines [15]. Another possible source of reactogenicity following the BNT162b2 mRNA COVID-19 vaccine can be attributed to the activation and presence of natural killer (NK) cells [63]. Reactogenicity following the BNT162b2 mRNA COVID-19 vaccine can be primarily linked to the activation and presence of NK cells, a critical role in the innate immune response, which has been observed to activate rapidly following mRNA vaccination. This activation has been associated with both the amplification of immune responses and the regulation of inflammation. Individuals with higher pre-vaccination NK cell cytotoxicity, a measure of their killing activity, reported higher symptom scores after the first dose of the vaccine. This suggests a direct correlation between NK cell activity and the inflammatory symptoms experienced post-vaccination. Specifically, the activation of NK cells can lead to the release of inflammatory cytokines and cytotoxic molecules, contributing to the local and systemic side effects observed after vaccination. mRNA itself also largely contributes to reactogenicity, which is driven by the activation of innate immune pathways. The activation of immune sensors triggers the release of pro-inflammatory cytokines, including TNF-α, IL-6, and interferons, which are largely responsible for symptoms such as fever, headache, and muscle pain [64]. Even FDA-approved commercial LNP vectors such as BNT162b2 have shown reactogenicity, as indicated by the safety data from phase 2/3 trials. Therefore, it is crucial to optimize both endosomal escape and reactogenicity to balance therapeutic efficacy with clinically acceptable levels of associated risks.

**Figure 6:**
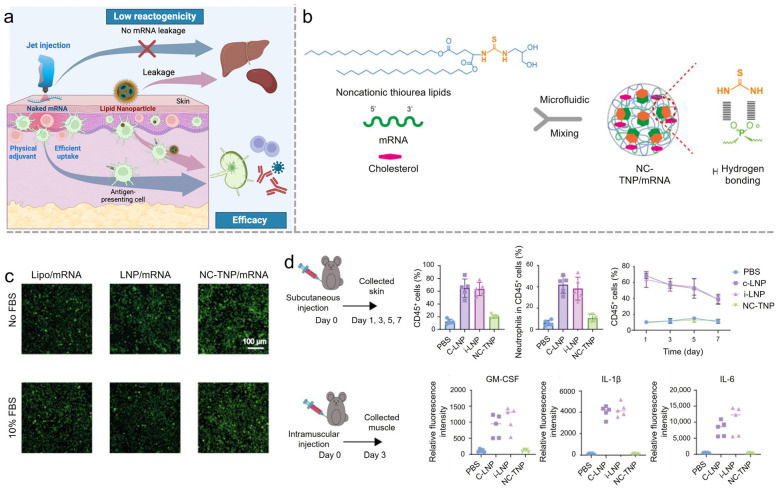
(**a**) Carrier-free mRNA vaccine induces robust immunity against SARS-CoV-2 in mice and non-human primates without systemic reactogenicity. Reproduced from ref. [65]. Copyright Cell Press. (**b**) Biomimetic noncationic LNPs for mRNA delivery. The red dashed circle, the NC-TNP binds to mRNA via hydrogen bonds. Reproduced from ref. [35]. Copyright NAS. (**c**) LNP and NC-TNP complexed with mRNA encoding EGFP incubated with DC2.4 cells to evaluate the EGFP protein expression by confocal microscopy images. Reproduced from ref. [35]. Copyright NAS. (**d**) Schematic illustration of the experiment design. C57BL/6 mice were subcutaneously or intramuscularly injected with c-LNP, i-LNP, and NC-TNP. After different times, the skin samples from the injection site were collected for analysis. Reproduced from ref. [35]. Copyright NAS.

Uchida et al. present an innovative approach to mRNA vaccine delivery that effectively addresses the critical issue of reactogenicity associated with traditional mRNA-LNP vaccines [65]. This method employs a needle-free, pyro-drive liquid jet injector (PYRO) to target the skin layer rich in antigen-presenting cells (APCs) such as dendritic cells, which are essential for initiating an immune response (Figure 6a). By delivering naked mRNA directly into these APCs, which then migrate to the lymph nodes to present the antigen, this approach ensures localized mRNA distribution, significantly reducing the risk of reactogenicity. The PYRO injector also acts as a physical adjuvant, inducing localized lymphocyte infiltration and proinflammatory responses at the injection site, thereby enhancing immunogenicity without the need for additional immunostimulatory adjuvants that can cause systemic inflammation. Studies on mice and non-human primates indicate the new solution induced strong antigen-specific antibody production and T-cell responses, comparable to traditional mRNA-LNP vaccines but with an improved safety profile. However, a potential limitation is the challenge of ensuring the stability and efficient cellular uptake of naked mRNA in the skin, as naked mRNA is highly susceptible to rapid degradation by extracellular RNases. This necessitates rapid internalization into cells, which may not always be achieved uniformly across different individuals or injection sites. Another solution, noncationic LNPs (NC-TNPs), to reduce inflammatory response was achieved by Deng et al. A noncationic lipid was synthesized by adding thiourea groups to achieve hydrogen bonding with mRNAs instead of relying on electrostatic force as traditional cationic lipid does [35] (Figure 6b). This new design reduces inflammatory effects by avoiding high cationic charge density. NC-TNPs also demonstrate higher transfection efficiency and fewer off-targeting effects than the conventional LNP system since it was proven that NC-TNPs targeted the spleen (Figure 6c,d).

## 5. Immunogenicity

**Figure 7:**
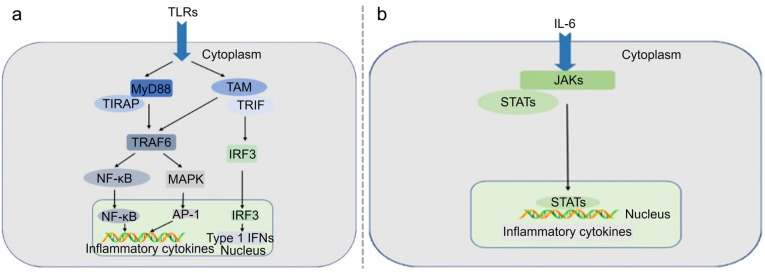
(**a**) MyD88-dependent and TRIF-dependent pathways signaling through TLRs activates intracellular signaling cascades that lead to nuclear translocation of AP-1 and NF-κB or IRF3. (**b**) Following IL-6 binding, the signal is transduced by a receptor to activate the JAKs, which then activate STATs. STATs are dephosphorylated in the nucleus, leading to the activation of downstream cytokines. Reproduced from ref. [68]. Copyright Impact Journals.

Immunogenicity refers to the ability of a substance to provoke an immune response, influencing both the efficacy and safety profile of LNPs. A robust immune response is essential for the effectiveness of vaccines, as it ensures the generation of a protective immune memory against pathogens. LNPs are recognized as foreign by the body’s immune system, which triggers innate immunity and subsequently affects adaptive immunity. Unmodified RNA can activate TLRs, specifically TLR3, TLR7, and TLR8, stimulating the innate immune system and enhancing the body’s initial defense mechanisms [66,67] (Figure 7a). This immune response can be beneficial by amplifying the effectiveness of mRNA vaccines, such as those developed for COVID-19, which have demonstrated robust protection and widespread use. In addition to the immunogenicity induced by RNA, the lipid components of LNPs can also be recognized by the NOD-like receptor proteins (NLRP3) inflammasome, IL-6 receptor, and Myeloid differentiation primary response 88 (MyD88) [68] (Figure 7b). These pathways help trigger immune responses and promote the production of inflammatory cytokines, essential for robust vaccine efficacy and a strong immune response. Still, excessive or uncontrolled activation can lead to adverse reactions. Thus, we want LNPs to stimulate these pathways to a controllable extent while balancing their activation to maximize therapeutic benefits and minimize potential side effects. Since mRNA is recognized as a foreign substance, it activates innate immune sensors such as TLR7, TLR8, and MDA5, inducing cytokine production and systemic inflammation. The immunogenicity related to the mRNA itself is not desirable for treatment, as the observed immunogenic effects are specifically due to the foreign nature of the cargo [69]. Despite modifications such as pseudouridine to reduce immunogenicity, partial immune activation still occurs [70]. These factors collectively contribute to both the immunogenic and inflammatory profiles of mRNA vaccines.

**Figure 8:**
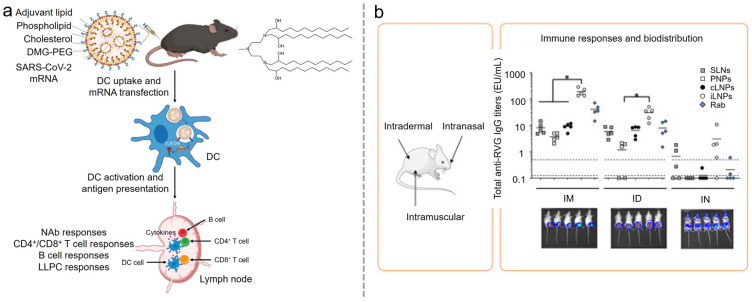
(**a**) Adjuvant lipidoid-substituted LNPs augment the immunogenicity of SARS-CoV-2 mRNA vaccines. Reproduced from ref. [36]. Copyright Nature Publishing Group. (**b**) The role of nanoparticle format and route of administration on self-amplifying mRNA vaccine potency. Reproduced from ref. [75]. Copyright Elsevier.

Enhancing immunogenicity is crucial when developing vaccines, especially for infectious diseases that require a robust immune response for effective protection. This is particularly important in cases involving highly virulent pathogens, those with high mutation rates, such as SARS-CoV-2, or in populations with weakened immune responses, such as the elderly or immunocompromised individuals. Enhancing immunogenicity ensures that the vaccine induces a strong and durable immune response, providing effective protection against the disease. This improved efficacy increases the ability of the vaccine to elicit strong and protective immune responses, including the production of neutralizing antibodies and the activation of T cells. Additionally, a more robust immune response can offer broader protection against multiple strains or variants of a pathogen and contribute to longer-lasting immunity, reducing the need for frequent booster doses. Ensuring high immunogenicity can make vaccines effective across diverse populations, including those with weaker immune systems. Mitchell et al. describe a strategy to enhance immunogenicity by incorporating adjuvant lipidoids into LNPs [36]. These adjuvant lipidoids possess Toll-like receptor 7/8 agonistic activity, which boosts the innate immune response, enhances mRNA delivery to cells, stimulates dendritic cell activation, and increases proinflammatory cytokine production (Figure 8a). This optimized approach induces strong Th1-biased cellular immunity, potent neutralizing antibodies, robust B cells, and long-lived plasma cell responses. Similarly, Anderson et al. discuss combining adjuvant properties with the ionizable lipid and mRNA itself [71]. This technique involves modified lipid formulations with intrinsic adjuvant properties, incorporating TLR agonists to directly activate dendritic cells, and improving antigen presentation, leading to a stronger adaptive immune response. Adjuvants work by creating a local immunocompetent environment at the injection site, stimulating innate immune responses, and influencing the type and strength of adaptive immune responses. For example, adding PAM3CSK4, a well-known lipopeptide adjuvant, to LNPs has been shown to improve survival rates, cellular responses, tumor growth inhibition, and humoral responses in mouse tumor models. Additionally, incorporating the TLR4 agonist lipopolysaccharide into LNPs has been found to boost CD8^+^ T-cell levels and antitumor activity [16]. Overall, the work discussed above highlights the importance of designing LNPs with inherent adjuvant properties to significantly enhance both innate and adaptive immune responses, thereby improving the overall immunogenicity of mRNA vaccines.

Although eliciting strong immune responses is desirable for vaccines, mitigating the immunogenicity of LNPs should be considered as well, especially for repeated administration. Elevated immunogenicity can precipitate adverse immune responses, including severe allergic reactions and autoimmune phenomena, thereby undermining therapeutic efficacy and compromising patient safety [16,71]. To address these challenges, sophisticated strategies are employed, such as adjusting the composition and physicochemical properties of LNPs. This involves fine-tuning the molar ratios of ionizable lipids, phospholipids, cholesterol, and PEGylated lipids. While PEGylated lipids enhance LNP stability and prolong circulation time, they can also elicit anti-PEG antibodies mentioned above, prompting immune reactions. Thus, incorporating cleavable PEG variants or biodegradable polymers can effectively mitigate this risk. For example, cleavable PEG-cholesterol derivatives such as PEG-CHMC, CHEMS, and CHST have shown promise in reducing the accelerated blood clearance phenomenon, which is often triggered by repeated administration of PEGylated LNPs [72]. Furthermore, optimizing the nanoparticle size and surface charge—where smaller, neutrally charged particles exhibit reduced immunogenicity and superior lymph node targeting—is critical [73]. The inclusion of specific adjuvants that modulate immune responses and the strategic selection of administration routes, such as IV, IM, subcutaneous (SC), intradermal (ID), or intranasal (IN), further refine immunogenic profiles (Figure 8b). For instance, IV administration of LNPs has been shown to induce high-level T-cell responses and profound antitumor efficacy compared to SC or ID routes. This is due to the ability of IV administration to mobilize substantial antigen-presenting cell pools in the spleen and other lymphoid tissues, leading to robust systemic immune responses. On the other hand, IN administration offers a noninvasive method that can generate both systemic and mucosal immunity by releasing IgA into the nasal cavity and intestinal tract [74,75,76,77]. These multifaceted approaches collectively enhance the therapeutic potential of LNPs by achieving an optimal balance of immunogenicity, thereby improving patient outcomes and broadening the clinical applicability of these advanced therapeutic modalities.

Despite the promising advancements in LNP for mRNA delivery, several drawbacks persist in the existing research requiring further investigation. One of the significant limitations is the inadequate understanding of the long-term immunogenicity of LNPs. Current studies predominantly concentrate on the short-term immune responses and immediate impacts on vaccine efficacy, leaving a considerable knowledge gap regarding the chronic effects and safety of LNPs, particularly for therapies requiring repeated administrations such as those for chronic diseases and genetic disorders. This gap underscores the need for comprehensive long-term studies that can elucidate the potential for immune tolerance, chronic inflammation, and the overall impact of repeated dosing over extended periods. Safety concerns related to the immunogenicity of LNPs also remain a critical issue. Adverse effects such as anaphylaxis, complement activation-related pseudoallergy, and potential autoimmune reactions are significant risks associated with LNP administration. While these adverse effects are well documented, the underlying mechanisms remain poorly understood, and current studies have yet to provide comprehensive strategies to mitigate these risks. This calls for in-depth mechanistic studies and the development of safer LNP formulations that can minimize these adverse effects while maintaining therapeutic efficacy. Furthermore, the exploration of alternative administration routes for LNPs is relatively limited. While intramuscular and intravenous routes are commonly used, there is insufficient research on the efficacy and safety of other potential routes, such as IN or SC injections. These alternative routes could offer distinct advantages but remain underexplored. Future research should investigate these routes more thoroughly to fully understand their potential benefits and drawbacks.

## 6. Summary and Outlook

The future development of LNPs should address a few challenges to be resolved. Several key strategies need to be implemented to enhance the targeting efficacy of LNPs for therapeutic applications. First, a primary goal in the development of LNP therapies is to achieve high levels of cell-specific expression. Future research should focus on achieving more precise organ targeting of LNPs, especially targeting specific cells within destined organs. Such fine-tuned targeting can enhance therapeutic efficiency and minimize side effects. Additionally, it is recommended that more effective in vitro evaluation platforms be established to assess the safety and side effects of LNPs, thus improving the translational potential for clinical applications. For example, compared to traditional in vivo models, in vitro platforms, such as microfluidic systems, offer lower costs and higher controllability to support LNP optimization. This involves ensuring that the therapeutic mRNAs are delivered primarily to the target cells, minimizing off-target effects that can lead to adverse outcomes. One approach to achieve this is using cell-specific promoters or targeting ligands that bind to receptors uniquely or predominantly expressed on the target cells. For instance, certain types of cancer cells express specific surface markers that can be targeted by ligands or antibodies conjugated to the LNPs. By exploiting these markers, LNPs can selectively deliver their cargoes to cancer cells, sparing healthy cells and reducing side effects. Optimizing the lipid composition and surface characteristics of LNPs is also a potential solution to enhance their ability to be taken up by specific cell types. This requires a deep understanding of the interactions between the nanoparticles and the cellular environment, including how the particles are recognized and internalized by different cell types. Research in this area is ongoing to identify the most effective combinations of lipids and target ligands for specific therapeutic applications. Secondly, active targeting can improve specificity and controlled release. Achieving super-selective cell expression through active targeting involves incorporating moieties on the surface of LNPs that specifically bind to receptors on the target cells [78,79]. However, the incorporation of these targeting moieties adds complexity to the manufacturing process. It requires precise control over the conjugation of ligands to the LNPs and the maintenance of their functional activity throughout the production and storage processes. Novel techniques and scalable production methods are needed to simplify the manufacturing of these targeted nanoparticles, making it feasible to produce them in large quantities for clinical use.

To enhance the biocompatibility of LNPs, investigating molecular structures that exhibit high biocompatibility is critical when designing ionizable lipids and other components. For instance, zwitterionic materials are known for their excellent compatibility with biological systems. Exploring new strategies for endosomal escape and cytosolic delivery based on organic chemistry is essential for achieving safe and efficient delivery because LNPs can be subject to lysosomal entrapment, where lipid components may degrade and compromise lysosomal membrane integrity, causing cytotoxicity. In recent years, the optimization and exploration of LNPs have involved extensive and complex screening processes, leading to the reporting of thousands of lipids. Establishing a streamlined regulatory framework for managing the vast array of LNPs and lipids plays a crucial role in handling LNP safety concerns. For example, the growing diversity of ionizable lipids, with lots of variants now available, has raised concerns about potential toxicity. Regulatory agencies must oversee these developments, ensuring that the manufacturing processes, component selection, and safety evaluation are standardized and thoroughly monitored.

## Figures and Tables

**Table 1:** Different LNP formulations targeting organs and their applications.

Key Lipids	Other Components	Targeted Organs and/or Cells	Applications	Ref.
5A2-SC8	Cholesterol, DMG-PEG, DOPE	Bone marrow (hematopoietic stem cells, leukemic cells, and mature blood cells)	Sickle cell disease and acute myeloid leukemia	[26]
BP-Lipid	Cholesterol, C14PEG2000, DOPE	Bone microenvironment and bone marrow (bone cells, cells of the hematopoietic and immune systems, fibroblasts, stromal cells, endothelial cells, monocytic lineage, B cell lineage, T cells, monocytes, granulocytes, B cells, and hematopoietic stem cells)	Skeletal diseases and age-related bone abnormalities (osteoporosis, osteoarthritis, osteomyelitis, and bone cancer)	[27]
C12-200	Cholesterol, DMG-PEG, DSPC	Liver (hepatocytes, endothelial cells, and Kupffer cells)	Deliver nucleic acids for gene therapy	[20]
DODAP (1,2-dioleoyl-3-dimethylammonium-propane)	Cholesterol, DMG-PEG, DSPC	Liver	Target the FVII gene in the liver	[28]
4A3-SC8	DOPE, Cholesterol, DMG-PEG, DOTAP	Lungs	Achieve durable gene correction for genetic lung diseases, such as cystic fibrosis	[29,30]
DOTAP (1,2-dioleoyl-3-trimethylammonium-propane)	Cholesterol, DMG-PEG, DSPC	Lungs and kidneys	Target the Tie2 gene in the lungs	[28]
5A2-SC8	DOPE, Cholesterol, DMG-PEG, 10% 18:1 PA (1,2-dioleoyl-sn-glycero-3-phosphate)	CD4^+^ and CD8^+^ T cells in spleen	B cell lymphoma	[31]
18PA (1,2-distearoyl-sn-glycero-3-phosphate)	Cholesterol, DMG-PEG, DSPC	Spleen	Target the CD31 gene in the spleen	[28]
Sazo/TAzo lipidoid	DOPE, Cholesterol, DMG-PEG	-	Free LNPs from endo-lysosomal compartments	[32]
6,6′-trehalose dioleate	DLin-MC3-DMA/SM-102, steroid, DOPE, DMG-PEG	-	Reduce organ toxicity	[33]
PolySarcosine	DMG-PEG, DODMA (1,2-dioleyloxy-3-dimethylaminopropane), DSPC, Cholesterol	-	Partially replace DMG-PEG to reduce hypersensitivity reactions and complement activation-related pseudo-allergy	[34]
Noncationic thiourea lipid	Cholesterol	-	Reduced reactogenicity caused by the inflammatory response	[35]
Adjuvant lipidoid (C12-TLRa)	Ionizable lipidoid, Phospholipid, Cholesterol, DMG-PEG	-	Enhance immunogenicity	[36]
